# Cooking can decrease mercury contamination of a mushroom meal: *Cantharellus cibarius* and *Amanita fulva*

**DOI:** 10.1007/s11356-017-8933-5

**Published:** 2017-04-06

**Authors:** Jerzy Falandysz, Małgorzata Drewnowska

**Affiliations:** Laboratory of Environmental Chemistry and Ecotoxicology, Gdańsk University, 63 Wita Stwosza Str, 80-308 Gdańsk, PL Poland

**Keywords:** Hazard decrease, Household treatment, Mercury, Mushrooms, Pollution

## Abstract

Mushrooms (*Cantharellus cibarius* and *Amanita fulva*) were blanched (parboiled) and pickled using different treatment conditions with the aim of carrying out the study into effect on removal of toxic mercury (Hg) accumulated in flesh. Blanching of fresh sliced *C. cibarius* caused leaching of Hg by approximately 15%, while loss of up to 35% was observed for sliced, deep-frozen fruit bodies. The rate of Hg leaching from the *C. cibarius* in practice was the same when blanched for 5 or 15 min irrespective of potable or deionized water used. Pickling of blanched *C. cibarius* with a diluted vinegar marinade had only a minor, if any, effect on removal of Hg and was without effect on blanched caps of *A. fulva*. Mercury was better extracted by boiling water from the fresh caps of *A. fulva* (56 ± 2% of the initial level in fresh caps) than from the fresh or frozen fruit bodies of *C. cibarius*. Total leaching rate of Hg from a pickled *C. cibarius* when fresh fruit bodies were processed was between 15 ± 5 and 37 ± 7% (median range 13–34%), and when deep-frozen fruit bodies were processed, it was between 37 ± 7 and 39 ± 8% (median range 34–39%). Pickling of the caps of *A. fulva* with diluted vinegar did not increase leaching of Hg. Blanching of mushrooms before future culinary use is a simple procedure recommended in reduction of contamination with Hg of cooked mushroom meal. Pickling had little if any effect on further removal of Hg from the initially blanched mushrooms.

## Introduction

Mushrooms foraged from the wild are traditional and popular organic food, which is considered rich in macro- and microelements and different organic compounds of nutritional and medicinal properties (Kalač 2016). They are valued by many around the world, while accessibility to good foraging areas, gourmet recipes and tradition, and intake per capita highly vary in regions of the world (Wang et al. 2014; Zhang et al. 2010). Estimated maximum intake is up to 20 ~ 30 kg fresh biomass per capita annually for the locals in Yunnan, China (Zhang et al. 2010).

The terrestrial mushrooms both saprobes and ectomycorrhizal due to specificities in their physiology may efficiently take-up from a polluted environment and accumulate in flesh different toxic chemical elements, e.g., Cd and Hg (Brzostowski et al. 2011; Falandysz et al. 2017; Gabriel et al. 2016; Kalač 2016). Mercury (Hg) is one of the metallic elements undesired in water media, human foods, and animal feeds, which as an environmentally hazardous compound is well accumulated in the fruit bodies by various species of mushrooms (Árvay et al. 2014; Falandysz 2016, 2017; Nasr and Arp 2011; Širić et al. 2017). Apart from an anthropogenic pollution with Hg, mushrooms can accumulate this element at great concentration also when grew in soils rich in geogenic Hg where bedrock is naturally enriched in Hg because of a geochemical anomalies (Kojta et al. 2015).

Cooking and industrial processing of mushrooms can have a pronounced impact on content and composition of compounds in a mushroom meals but the data are generally limited for Hg or a wide spectrum of the metallic elements and metalloids (Biekman et al. 1996; Coskuner and Özdemir 1997; Svoboda et al. 2002; Vetter 2003, Źrodlowski 1995). Recently, a polystyrene-g-2-adenine chelating resin was developed and used to preconcentrate and detect the concentration of Hg ions in edible mushrooms (Li et al. 2016) or selective removal of copper with polystyrene-1,3-diaminourea chelating resin (Shen et al. 2016), which technologies in principle is without any future in application for foods.

Mushrooms can be cooked in different manner, and the cooking books gave many recipes. Some of the treatments are of more general or universal nature, e.g., a short time (5–15 min) boiling (blanching; parboiling) or boiling for a longer time, while other can be specific for a species of mushroom or a dish. Blanching is a radical manner, because of excess of boiling water used, which causes fruit body dehydration and shrinkage, and denaturation, hydrolysis, and dissolution of the building and other organic constituents of mushroom. Blanching efficiently removes hazardous constituents such as alkali element—radiocaesium (^137^Cs)—from contaminated mushrooms (Skibniewska and Smoczyński 1999). Blanching also significantly removes from mushrooms many bio-elements, which was little studied so far, while apart from radiocaesium, it can also decrease content of the toxic metallic contaminants such as cadmium (Cd), lead (Pb), and Hg (Svoboda et al. 2002). This study investigates the potential effect of blanching and pickling in removal of Hg from a whole fruit bodies of chanterelle *C. cibarius* Fr. and from the caps of tawny grisette with current name in Latin as *A. fulva* Fr., and several synonyms: *Agaricus fulvus* Schaeff., *A. fulva f. alba* (Courtec.), *Amanita vaginata var. fulva* (Fr.) Gillet, *Amanitopsis fulva* (Fr.), *Amanitopsis fulva F. alba* Courtec., *Amanitopsis fulva* (Fr.) W.G. Sm., *Amanitopsis vaginata* var. *fulva* (Fr.) Sacc., and *Vaginata fulva* (Fr.) A.H. Sm. (Species Fungorum 2017).

Blanching of *C. cibarius* and other mushrooms before further cooking (common frying, frying with eggs, freezing, and further frying with eggs or stewing, using for soup or pickling) is common procedure in the Polish kitchen.

## Materials and methods

### Mushrooms and other products

Five sets of matured fruit bodies of *C. cibarius* samples (from 267 to 358 fruit bodies in a pool per site) and one set of 60 matured fruit bodies of *A. fulva* (only caps selected) were used in the study. *C. cibarius* were collected from five background (unpolluted) areas: (i) forest near the Jastrzębia Góra, coordinates 54.8312° N, 18.3129° E; (ii) Łapino, coordinates 54.3000 °N, 18.4333 °E; (iii) Kościerzyna forests, coordinates 54.0415° N, 18.2392° E; (iv) the Darżlubska Wilderness, coordinates 54.6747° N, 18.2577° E; and (v) Tucholskie Pinewoods, coordinates 53.8152° N, 17.5648° E in the north-central region of Poland in 2012–2014. *A. fulva* was collected from a forest nearby to the location Łapino (coordinates 54.3000° N, 18.4333° E) in the Kolbudy Forest Inspectorate of Pomerania in 2011.

Fruit bodies of *C. cibarius* were, in aim of the removal of adhering debris, rinsed with cold tap water and drained. Next, each fruit body in a sample set was divided into four or three parts (vertical cuts using a plastic knife), which were pooled and further subsampled and treated accordingly—dried, frozen, blanched, and blanched/pickled (Table 1). The caps of *A. fulva* were in situ cleaned from any adhering foreign debris. Next, they were divided into quadrants, which were pooled (four samples) and further divided for a subsamples and treated accordingly (Table 1).

**Table 1:** Data on mercury concentrations (mg kg^−1^ dry biomass) and mercury leaching rate (%) from household treated *C*. *cibarius* and *A. fulva*

Parameter	Species	*C. cibarius*					*A. fulva*
	Sample size	7 (311)^a^	7 (267)	7 (331)	8 (358)	7 (298)	4 (60)
	Status	Fresh	Fresh	Fresh	Deep frozen	Deep frozen	Fresh
Hg	Mean ± S.D.	0.032 ± 0.004	0.017 ± 0.003	0.034 ± 0.011	0.041 ± 0.011	0.033 ± 0.009	0.23 ± 0.02
	Range	0.027–0.037	0.013–0.021	0.027–0.053	0.032–0.057	0.022–0.050	0.21–0.25
	Median	0.030	0.017	0.028	0.035	0.031	0.22
	Treatment	Blanching	Blanching	Blanching	Blanching	Blanching	Blanching
	Water	Potable	Potable	Deionized	Potable	Deionized	Deionized
	Time (min)	5	15	15	15	15	15
Hg	Mean ± S.D.	0.028 ± 0.003	0.015 ± 0.003	0.030 ± 0.009	0.027 ± 0.006	0.021 ± 0.006	0.099 ± 0.010
	Range	0.023–0.031	0.012–0.019	0.024–0.045	0.021–0.036	0.015–0.033	0.087–0.11
	Median	0.027	0.014	0.026	0.024	0.021	0.098
Loss *vis*. fresh or deep-frozen mushrooms (%)	Mean ± S.D.	13 ± 5	13 ± 5	12 ± 2	33 ± 6	36 ± 6	56 ± 2
	Range	9.0–20	8.0–22	9.0–15	20–43	31–50	55–58
	Median	10	11	11	34	34	56
	Treatment	Pickling^b^	Pickling	Pickling	Pickling	Pickling	Pickling
Hg	Mean ± S.D.	0.025 ± 0.002	0.014 ± 0.003	0.029 ± 0.007	0.025 ± 0.004	0.020 ± 0.005	0.11 ± 0.01
	Range	0.022–0.028	0.0095–0.018	0.023–0.041	0.021–0.032	0.015–0.030	0.10–0.13
	Median	0.024	0.013	0.026	0.023	0.019	0.11
Loss *vis*. blanched mushrooms (%)	Mean ± S.D.	10 ± 7^c^	8.0 ± 6.4^d^	4.2 ± 3.7^e^	6.7 ± 7.1^f^	8.5 ± 9.3^g^	0
	Range	0–22	0–18	0–9.6	0–18	0–24	0
	Median	9.0	6.7	3.9	6.2	7.8	0
Total loss (%)	Mean ± S.D.	21 ± 6	19 ± 7	15 ± 5	37 ± 7	39 ± 8	50 ± 3
	Range	16–29	10–33	7.0–22	29–49	29–50	47–53
	Median	22	18	13	34	39	51

^a^Number of composite samples and total number of fruiting bodies/caps (in parentheses)

^b^Blanched mushrooms were pickled

^c^In one sample, an increase by 3%

^d^In one sample, an increase by 4%

^e^In two samples, an increase by 2 and 3%

^f^In one sample, an increase by 3%

^g^In three samples, an increase by 4, 5, and 21%

Deionized water free of Hg, potable bottled water (Fonte spring from the location Nieszawa in Poland) free of Hg, and commercial spirit vinegar were used as media in the blanching and pickling experiments. The spirit vinegar (10% acetic acid solution) free of Hg (<5 ng L^−1^) in glass bottle (0.5 L) with an aluminum screw cap and polyethylene gasket used for preparation of marinade was bought in a grocery shop.

### Conventional drying

From each sample set of a freshly sliced fruit bodies or caps, the subsamples were selected. They were placed into plastic trays of an electrically heated commercial dryer (dehydrator for vegetables; model MSG-01; MPM Product, Milanówek, Poland) and dried at 65 °C to constant mass. Dried fungal materials were ground using a porcelain pestles and mortars that were cleaned by hand washing using laboratory brush, deionized water, and detergent, and further rinsed with distilled water and dried in an electrically heated laboratory dryer at 105 °C. Ground fungal materials were transferred into a screw-capped plastic tubes (VWR®, Ultra High Performance, 15 mL) and closed, and the tubes were packed into polyethylene bags and kept sealed in dry and clean condition in a storage room until analysis. Dried and ground fungal materials were considered as the reference samples for Hg determinations.

### Deep freezing

Two subsamples of *C. cibarius* were further divided into portions and kept frozen at −20 °C for 1 month. The subsamples of the frozen mushrooms were freeze dried (lyophilizer model LYOVAC GT2; Steris, Germany). The freeze-dried mushrooms were ground in the same way as those which were dried conventionally and, in a form of a powder, were further transferred into a screw-capped plastic tubes, which were kept closed in sealed polyethylene bags until analysis.

### Blanching

Fresh or frozen sliced fruit bodies or caps were blanched respectively for 5 to 15 min in gently boiling deionized water (150 mL) in a glass beakers or in potable water in a stainless steel pot. The ratio of the mushroom matter to water was 1:5 (*w*/*w*). The blanched mushrooms were next drained using plastic colander, freeze dried, ground, transferred into a screw-capped plastic tubes, and kept for further analyses or were pickled to examine impact of pickling (Table 1).

### Pickling

Freshly blanched *C. cibarius* and *A. fulva* samples directly after draining were pickled using a vinegar-based marinade. Marinade was made by dilution of vinegar with tap or deionized water in proportion 1: 4. Mushrooms were pickled in glass beakers (150 mL). Beakers filled with mushrooms and marinade were tightly sealed with a plastic foil from the top and kept in room temperature for 1 month. Next, mushrooms were drained and subsamples were freeze dried and ground, using porcelain pestle and mortar, and powdered samples were packed into a screw-capped plastic tubes (VWR®, Ultra High Performance, 15 mL). Tubes were further packed into a foil bag, which was sealed, and kept until analysis in dry and clean condition in a storage room.

### Mercury analysis

The reagents used in this study were of analytical reagent grade. Deionized water was used for the preparation of the solutions. Mercury standard solution of 1.0 mg mL^−1^ was obtained from the 10 mg mL^−1^ standard stock solution. Blanks and 3, 5, 10, 15, and 20 μL (low mode) and 25, 50, 100, 150, and 200 μL (high mode) of 1.0 mg mL^−1^ Hg standard solutions were injected into the analyzer for the construction of calibration curves.

Mercury content of all fungal and blank samples was determined using cold-vapor atomic absorption spectroscopy (CV-AAS) by a direct sampled material thermal decomposition coupled with gold wool trap of Hg vapors and its further desorption and quantitative measurement at wavelength of 253.7 nm. The analytical instrument used was mercury analyzer (MA-2000, Nippon Instruments Corporation, Takatsuki, Japan) equipped with autosampler and operated respectively at low (3 to 20 ng Hg per sample) and high (25 to 150 ng Hg per sample) mode (Falandysz et al. 2012).

Analytical control and assurance quality (AC/AQ) was assessed through analysis of blank samples and two certified reference materials: CS-M-2 (dried mushroom powder *Agaricus campestris* for which declared Hg content was 0.164 ± 0.004 mg kg^−1^ dm) and CS-M-4 (dried mushroom powder *Leccinum scabrum* for which declared content was 0.465 ± 0.024 mg kg^−1^ db)—both produced by the Institute of Nuclear Chemistry and Technology, Warsaw, Poland. Our result for CS-M-2 was 0.164 ± 0.006 mg kg^−1^ db (*n* = 6; mean recovery 101%) and for CS-M4 was 0.448 ± 0.010 mg kg^−1^ db (*n* = 6; mean recovery 98%).

## Results and discussion

### Fresh mushrooms

Mercury concentrations in the composite samples of *C. cibarius* subjected for a household treatment were from 0.017 ± 0.003 to 0.041 ± 0.011 mg kg^−1^ dry biomass (db) on the average, and total range was 0.013–0.057 mg kg^−1^ db (Table 1). The results showed on a small contamination of *C. cibarius* with Hg, while the values agreed with data reported for this species foraged from the background forested areas in Poland (Falandysz et al. 2012).

Mercury concentration in *A. fulva* was at 0.23 ± 0.02 mg kg^−1^ db (Table 1). This result agreed with the values reported for *A. fulva* foraged across Poland and also with data for *Amanita vaginata* (Bull.) Lam., which is a mushroom from the same section as *A. fulva* (Drewnowska et al. 2014; Falandysz and Drewnowska 2015).

### Blanched mushrooms

Fresh fruit bodies of *C. cibarius* when blanched for 5 or 15 min in potable or deionized water dropped Hg concentration by 12 ± 2 to 13 ± 5% (total range 8.0–22%). Fruit bodies which were initially deep frozen and further blanched dropped Hg by 33 ± 6 to 36 ± 6% (total range 20–50%). Fresh caps of *A. fulva* during blanching for 15 min dropped Hg concentration from 0.23 ± 0.02 to 0.098 ± 0.010 mg kg^−1^ db—decrease by 56 ± 2% (Table 1).

The rates of Hg leaching from *C. cibarius* were in practice the same when blanched for 5 or 15 min and in potable or deionized water. A rate of Hg leaching from the caps of *A. fulva* was higher than the 10% drop reported for halves of a whole fruit bodies of this mushroom blanched for 10 min in boiled tap water (rich in calcium) (Falandysz and Drewnowska 2015).

Clearly, in the blanching experiments performed in this study, it was harder to remove Hg sequestered in flesh by *C. cibarius* than that from the quadrants of the caps of *A. fulva*. Results on the leaching rates of Hg obtained in this study were roughly similar with those reported from the experiments with blanched mushroom *Imleria badia* (Fr.) Vizzini (former name *Xerocomus badius* (Fr.) E.-J. Gilbert). Namely, sliced fresh fruit bodies of the *I. badia* when blanched in the salted water for 15 min lost ~15% of Hg, while when were blanched a deep frozen mushrooms, a drop of Hg was ~22% (Svoboda et al. 2002).

### Pickled mushrooms

Samples of blanched mushrooms from the particular experiments were also pickled under conditions given in previous section. Pickling is a common household treatment for *C. cibarius* and caps of *A. fulva*. When mushrooms are prepared for home use or commerce, they can be pickled in a vinegar-based marinade alone or with added table salt, carrot, onion, and spices. No salt, vegetables, or species were added to marinade in this study.

Pickling had only a minor effect or was without effect on leaching of Hg from the blanched mushrooms. In the case of *C. cibarius*, some portion of Hg leached out of the sliced fruit bodies, e.g., by around 2–3%, while for some samples, there was no leaching but a slight increase in Hg content (when data were normalized to dry biomass content). A reason for lack of decrease of Hg content in blanched fungal material when they were pickled can be a selective leakage of some organic compounds free or with low content of Hg but in parallel was no leaching or only a weak leaching of Hg. This can result in a slight increase in content of the elements (Hg) which does not leach or leach in lower rates than the bulk organic materials of the fruit bodies. No loss of Hg from the caps was noted for pickled *A. fulva* when related to the blanched caps (Table 1).

In this study, fresh and sliced fruit bodies of *C. cibarius* when blanched lost on average 36% of biomass, while when they were further pickled, they lost in total 40% of the original biomass. Frozen *C. cibarius* when blanched lost 62% of biomass, while when further pickled, lost 74% of biomass. Blanching of *Agaricus bisporus* (J.E. Lange) Imbach caused a decrease of the biomass of the fruit bodies by 30% due to a partial loss of water and water soluble constituents, while for *Grifola frondosa* (Dicks.) Gray and *Flammulina velutipes* (Curtis) Singer, decrease was in the range 11–16% (Dikeman et al. 2005).

Total leaching rate of Hg for *C. cibarius* was between 15 ± 5 and 21 ± 6% when fresh mushrooms were treated (blanched and pickled) and between 37 ± 7 and 39 ± 8% when frozen mushrooms were treated. For blanched or blanched and pickled caps of *A. fulva*, drop of Hg was between 50 ± 3 and 56 ± 2%.

This is known that radiocaesium and some other compounds leak out of the fruit bodies at higher rate when the fungal cells were more or less broken, because of a specific treatment of fruit bodies, which were deep frozen, sliced, or powdered than a whole fresh or a little defragmented (sliced) fruit bodies (Kenigsberg et al. 1996; Svoboda et al. 2002). An observed difference in the leaking rates of Hg between *C. cibarius* and *A. fulva* can be also because of a difference in the texture of the flesh of the fruit body between both species, which is more fleshy and firm for *C. cibarius* while much more fragile and softer for *A. fulva*.

Mercury in champignon *A. bisporus* cultivated in compost fortified with Hg salt (^203^Hg) was noted to be bound largely in high-molecular-mass proteins, and oyster mushroom *Pleurotus ostreatus* was in protein fraction of 17–45 kDa (Lasota and Florczak 1991). Evidently, during blanching of *I. badia*, Hg leached at much lower degree than radiocaesium (^137^Cs) (Skibniewska and Smoczyński 1999; Svoboda et al. 2002). Radiocaesium ^137^Cs and ^134^Cs (and stable caesium, ^133^Cs) forms weaker bonds in salts and can be largely in protoplasm than other parts of the cells in the fruit bodies, while not in proteins like is an inorganic Hg. Inorganic Hg is a major (> 90%, on the average) Hg compound in mushrooms. Mercury can form more firm bonds than caesium and especially with sulfhydryl groups of proteins or can form other sparklingly soluble and more difficult for leaching compounds in mushrooms.

Summing up, the Hg was better extracted by boiling water from the caps of *A. fulva* than from the fruit bodies of *C. cibarius*. Blanching of fresh and sliced fruit bodies of *C. cibarius* caused leakage of Hg by around 15%, while blanching of deep-frozen and sliced fruit bodies caused leakage of Hg by around 35%. The rate of Hg leaching from the *C. cibarius* in practice was the same when blanched for 5 or 15 min irrespective of potable or deionized water used. Blanching of the quadrants of fresh caps of the *A. fulva* causes loss of Hg by around 56%. Pickling had only little impact if any on leaching of Hg from the blanched fruit bodies of *C. cibarius* or caps of *A. fulva*. Lack of any significant leaching of Hg from the blanched mushrooms when pickled using diluted vinegar can imply on its occurrence in mushrooms large in form of a sparklingly soluble compounds of a type HgSe, HgS and//or bonds with sulphydryl groups (−SH) in macromolecules (proteins), which were not degraded and leached by boiling water. These compounds can be weakly leachable from pickled mushrooms in human gut, while blanching can remove from the fruit bodies a large portion of Hg attached with soluble or colloidal fractions of the flesh which can be discarded with the waste water. Hence, in practice, blanching of mushrooms before future culinary use is a simple procedure recommended in reduction of Hg contamination in foraged and cultivated mushrooms and which can be good also for mushrooms harvested from grounds subjected for mycoremediation because of Hg pollution. Pickling had little if any effect on further removal of Hg from the initially blanched mushrooms.
